# Volumetric optoacoustic neurobehavioral tracking of epileptic seizures in freely-swimming zebrafish larvae

**DOI:** 10.3389/fnmol.2022.1004518

**Published:** 2022-09-13

**Authors:** Çağla Özsoy, Adriana L. Hotz, Nicolas N. Rieser, Zhenyue Chen, Xosé Luís Deán-Ben, Stephan C. F. Neuhauss, Daniel Razansky

**Affiliations:** ^1^Faculty of Medicine, Institute of Pharmacology and Toxicology and Institute for Biomedical Engineering, University of Zurich, Zurich, Switzerland; ^2^Department of Information Technology and Electrical Engineering, Institute for Biomedical Engineering, ETH Zurich, Zurich, Switzerland; ^3^Department of Molecular Life Sciences, University of Zurich, Zurich, Switzerland

**Keywords:** calcium imaging, fluorescence microscopy, optoacoustic tomography, photoacoustics, functional neuroimaging, epilepsy, Danio rerio

## Abstract

Fast three-dimensional imaging of freely-swimming zebrafish is essential to understand the link between neuronal activity and behavioral changes during epileptic seizures. Studying the complex spatiotemporal patterns of neuronal activity at the whole-brain or -body level typically requires physical restraint, thus hindering the observation of unperturbed behavior. Here we report on real-time volumetric optoacoustic imaging of aberrant circular swimming activity and calcium transients in freely behaving zebrafish larvae, continuously covering their motion across an entire three-dimensional region. The high spatiotemporal resolution of the technique enables capturing ictal-like epileptic seizure events and quantifying their propagation speed, independently validated with simultaneous widefield fluorescence recordings. The work sets the stage for discerning functional interconnections between zebrafish behavior and neuronal activity for studying fundamental mechanisms of epilepsy and *in vivo* validation of treatment strategies.

## Introduction

Epilepsy is a brain disorder characterized by pathological neuronal dynamics and spontaneous recurrent seizures resulting from imbalance between excitatory and inhibitory synaptic transmission (Fisher et al., 2014). Animal models have significantly contributed to advancing our understanding on the basic mechanisms of epilepsy, which is essential to develop more effective anti-epileptic drugs (Parker et al., 2011; Löscher, 2017). Particularly, zebrafish is an excellent translational model to investigate epilepsy and other neurological disorders (Stewart et al., 2012; Yaksi et al., 2021). Zebrafish produce a large offspring developing rapidly and experience similar neuropharmacological effects of drugs as humans (Wyatt et al., 2015; Fontana et al., 2018). Optical transparency of larval zebrafish further enabled following complex behaviors associated to evolutionary-conserved and decision-making neuronal networks (Patton et al., 2021). Classical convulsant drugs such as pentylenetetrazole (PTZ) and kainic acid (KA) have been widely used for evoking seizure-like behavior in zebrafish and represent a suitable model of acute epileptic seizures (Gawel et al., 2020). While such drugs are ideal for targeting intact healthy brains, genetic models with recurrent spontaneous seizures may even better represent the long process of epileptogenesis. For example, the model of glutamate transporter EAAT2a-deficiency in the zebrafish offers a new platform for assessing chronic epilepsy (Hotz et al., 2022). The lowered threshold for spontaneous epileptic seizures in the *eaat2a* mutant zebrafish model of perturbed glutamate clearance also facilitates triggering seizures by sensory light stimulation (Yaksi et al., 2021; Myren-Svelstad et al., 2022).

Epileptic seizures in zebrafish have traditionally been analyzed by either tracking behavioral changes in freely-swimming larvae (Baraban et al., 2005; Winter et al., 2008) or by recording brain activity in immobilized animals (Baraban et al., 2005; Burrows et al., 2020). The development of genetically encoded calcium indicators (GECI), particularly GCaMP proteins, has enabled new means of investigating neuronal activity (Akerboom et al., 2012; Chen et al., 2013). GECIs further facilitated the development of new fluorescence (FL) imaging solutions, e.g., based on light-sheet or light-field microscopy for volumetric imaging of zebrafish brain in action (Ahrens et al., 2013; Prevedel et al., 2014; Cong et al., 2017). Simultaneous imaging of brain activity and behavior in freely-swimming fluorescent reporter fish has also been reported by employing an optical tracking system (Symvoulidis et al., 2017), albeit the measurements were limited to planar (2D) fluorescence observations (Johnson et al., 2020).

Hybrid optoacoustic (OA) imaging methods can overcome the spatiotemporal performance and field-of-view (FOV) restrictions associated with optical microscopy. State-of-the-art optoacoustic tomography (OAT) systems based on spherical matrix arrays have been reported to achieve volumetric imaging rates in the kilohertz range while covering extended FOV on a centimeter scale (Özbek et al., 2018; Özsoy et al., 2021). The method has further been shown to provide direct readings of neuronal activity by exploiting calcium-dependent absorptivity changes in the spectrum of GECIs (Deán-Ben et al., 2016). In this work, we report on real-time 3D imaging of calcium (Ca^2+^) transients in freely-swimming genetically modified *Tg(elavl3:GCaMP5G);nacre*^–/–^ zebrafish larvae expressing the GCaMP5G indicator with volumetric OAT. The high acquisition speed enables capturing ictal-like epileptic seizure events in EAAT2a mutants, which can be used to unveil functional interconnections between zebrafish behavior and neuronal activity. Simultaneous widefield FL recordings were also performed to independently validate the OAT imaging results and quantify the seizure propagation speeds in both modes.

## Materials and methods

### Animal model, maintenance, and handling

Brain excitability is tightly controlled by balancing excitatory and inhibitory inputs with the excitatory amino acid transporter 2 (EAAT2) playing a key role in this process. The EAAT2 is expressed in astroglia in the central nervous system (CNS) and regulates synaptic transmission by removing the extracellular glutamate (Danbolt, 2001). Therefore, the hyperexcitability promoted by *eaat2a*-deficiency allows investigating seizure initiation and propagation in response to light stimulation (Hotz et al., 2022; Myren-Svelstad et al., 2022). To this end, brain-wide increased neuronal excitability in *eaat2a*^–/–^ zebrafish (*Danio rerio*) has been demonstrated *via* neuronal responses to transient light flashes (Hotz et al., 2022). CRISPR/Cas9-mediated *eaat2a* mutants (Hotz et al., 2022) were kept under standard conditions (Mullins et al., 1994). Homozygous *eaat2a*^–/–^ mutants and control siblings in the *Tg(elavl3:GCaMP5G);nacre*^–/–^ background expressing GCaMP5G in all neurons (Akerboom et al., 2012) were generated by incrosses of adult *eaat2a*^+/–^ fish. Zebrafish embryos were kept at 28°C with 10:14 h dark-light cycles. They were raised in E3 medium (5 mM NaCl, 0.17 mM KCl, 0.33 mM CaCl_2_, 0.33 mM MgSO_4_) containing 0.003% 1-phenyl-2-thiourea (PTU) to reduce pigment formation in the eye (Karlsson et al., 2001). At 5 days post-fertilization (dpf), a total of *n* = 20 *eaat2a*^+/–^ or ^+^*^/^*^+^ and *n* = 20 *eaat2a*^–/–^ zebrafish larvae were used for the experiments. All procedures involving animals and their care were conducted in full compliance with the institutional guidelines and under approval from the Cantonal Veterinary Office of Zurich (BNr-ZH150).

### Dual-mode optoacoustic fluorescence imaging system

**FIGURE 1:**
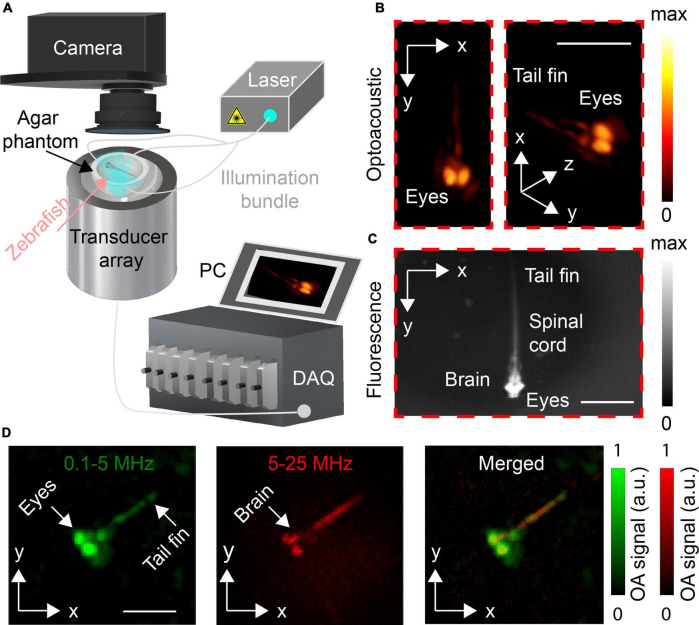
**(A)** Lay-out of the experimental system. **(B)** Volumetric optoacoustic tomography (OAT) image of the 5 dpf control zebrafish larva. Maximum intensity projections (MIPs) for top (left) and tilted (right) views are shown. **(C)** Corresponding widefield fluorescence (FL) image of the same zebrafish larva. **(D)** MIPs along the z axis of the OAT images reconstructed with frequency bands of 0.1–5 and 5–25 MHz. The merged image is also shown. Scalebars–2 mm.

The layout of the concurrent OAT and widefield FL imaging system is illustrated in Figure 1A. OAT imaging was performed with a custom-made spherical matrix array transducer comprising of 512 individual elements with detection bandwidth extending up to 35 MHz, as described in detail elsewhere (Deán-Ben et al., 2017). The system provides a nearly isotropic 3D resolution of <30 μm effectively covering a FOV of 5 × 5 × 5 cm^3^. The OA signal excitation was performed with an optical parametric oscillator (OPO)-based laser (EVO I OPO 355 nm broadband, Innolas GmbH, Krailling, Germany) providing short pulses at 25 Hz pulse repetition frequency tuned to 488 nm, the peak absorption wavelength of GCaMP5 proteins. The laser light was delivered to the imaged object from multiple directions by means of a multi-arm fiber bundle, resulting in ∼5 mJ/cm^2^ light fluence within the imaged region. The generated OA signals were digitized with a custom-made data acquisition unit (DAQ, Falkenstein Microsysteme, Taufkirchen, Germany) triggered with the Q-switch output of the OPO-based laser. The digitized signals were transferred to a PC *via* 1 Gbit/s Ethernet connection.

To enable imaging of behaving zebrafish larvae, a solidified agar medium was placed on top of the spherical array and a 5 mm diameter groove was carved in the agar and filled with E3 medium (5 mM NaCl, 0.17 mM KCl, 0.33 mM CaCl_2_, 0.33 mM MgSO_4_). 5 dpf zebrafish larvae were allowed to freely swim inside the groove. The volume between the active surface of the array and the agar mold was filled with deionized water to ensure acoustic coupling The larvae were given 1 min to adapt before the imaging session has commenced. Light-induced stimulation (488 nm) was used for initiating seizure-like activity in the zebrafish larvae. Turning on the excitation laser (488 nm) for FL imaging resulted in enhanced seizure-like activity in hyperexcitable *eaat2a*^–/–^ larvae (Hotz et al., 2022). Concurrent OAT and widefield FL recording was conducted for 300 s.

### Optoacoustic image reconstruction and processing

**FIGURE 2:**
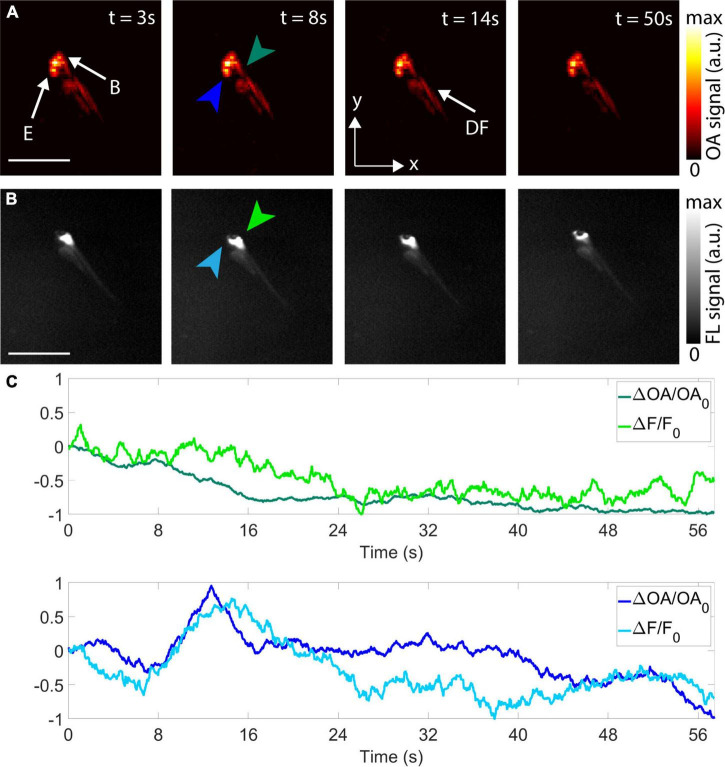
Imaging of epileptic seizure propagation in 5 dpf zebrafish larvae. **(A)** Optoacoustic tomography (OAT) images at 3, 8, 14, and 50 s after the beginning of the acquisition–maximum intensity projections (MIPs) along the z direction are shown. E, eyes; B, brain; DF, dorsal fin. **(B)** Corresponding FL images. **(C)** Temporal profiles of the differential OA [ΔOA/OA_0_ = (OA–OA_0_)/OA_0_, green and blue plots] and the differential FL [ΔF/F_0_ = (F–F_0_)/F_0_, light green and light blue plots] signals for two different points in the hindbrain (green and light green arrows) and forebrain (blue and light blue arrows). Scalebars–2 mm.

**FIGURE 3:**
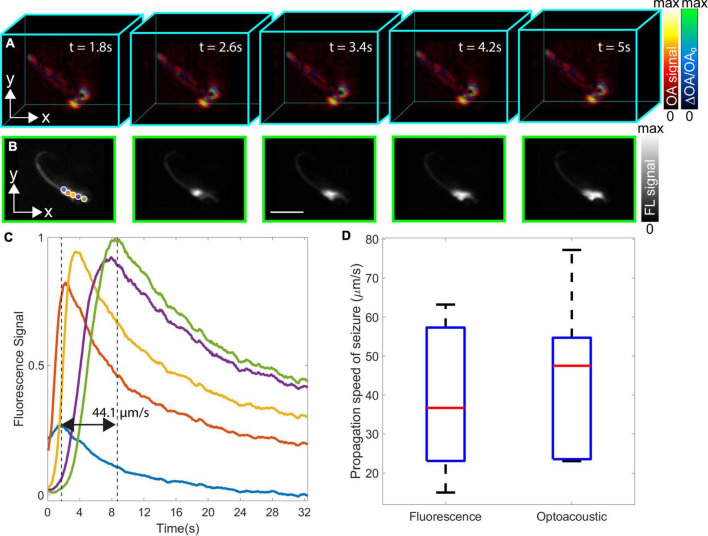
Dual-modality volumetric optoacoustic tomography (OAT) and fluorescence (FL) imaging of neuronal activation in the *eaat2a*^–/–^ mutant zebrafish larva and estimation of epileptic seizure propagation speed. **(A)** Relative optoacoustic (OA) signal changes (ΔOA/OA_0_) from five consecutive time points superimposed on the normalized volumetric OAT image. Light-induced stimulation was initiated at *t* = 0 s. **(B)** The corresponding time-lapse FL image sequence. **(C)** Spatio-temporal plots representing seizure propagation in five different points labeled in blue, orange, yellow, purple, and green colors in panel **B**. The FL signal peak of blue and green plots corresponds to the 3 and 8 s time points with the speed of seizure propagation calculated as 44.1 μm/s. **(D)** Boxplots of the propagation speed of epileptic seizures calculated from the *eaat2a*^–/–^ mutant zebrafish larvae (5 dpf) (*n* = 7) [FL; mean: 39.6, mean ± σ: (58.6001, 20.5713) μm/s versus OA; mean: 43.5, mean ± σ: (64.3477, 22.5951) μm/s]. Scale bar, 2 mm.

**FIGURE 4:**
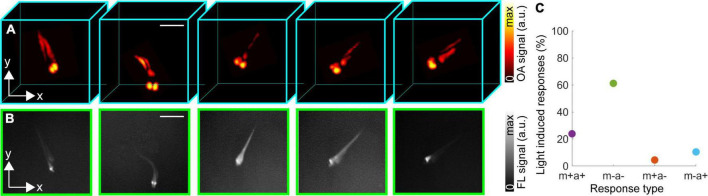
Aberrant swimming activity of the freely moving *eaat2a*^–/–^ mutant zebrafish larva following light-induced stimulation at 488 nm. **(A)** Volumetric optoacoustic (OA) images at 0, 0.2, 1.2, 2.6, and 8 s after the stimulation beginning. **(B)** Corresponding fluorescence (FL) images. Scalebars–3 mm. **(C)** Light induced different types of responses (%) following 67 total stimulations in 12 *eaat2a*^–/–^ mutant zebrafish larvae.

Time-lapse volumetric OAT images covering 7 × 7 × 7 mm^3^ volume consisting of 128 × 128 × 128 voxels^3^ were reconstructed using a GPU-accelerated filtered back-projection algorithm (Deán-Ben et al., 2013; Figure 1B). Prior to reconstruction, OA signals were band-pass filtered between 0.1 and 25 MHz. The broadband OA signals were separated into two different ultrasound frequency bands (the low 0.1–5 MHz band and the high 5–25 MHz band) using second order band-pass Butterworth filter in order to better differentiate large and fine zebrafish structures in the reconstructed images. The OA images obtained from the low and high frequency bands were normalized between 0 and 1 and visualized with different colors, namely, red and green (Figure 1D). OA signals were band-pass filtered with the selected frequency bands, i.e., 5–25 MHz (Figure 2A), 0.1–25 MHz (Figure 3A), and 0.1–6 MHz (Figure 4A) to clearly illustrate the activation changes while providing a suitable anatomical reference. For each acquired time-lapse image sequence, the baseline values (OA_0_) were calculated by averaging the first ten time points at the beginning of the OA image sequence. Differential OA signals with respect to the pre-seizure levels, namely, ΔOA/OA_0_ = (OA−OA_0_)/OA_0_, were calculated for different voxels in the hindbrain and forebrain regions to identify the GCaMP5-related activity. 3D neuronal activation maps of *eaat2a*^–/–^ mutant zebrafish larvae were generated by first subtracting the reference frame. Each resulting sequence was subsequently normalized, and a mask was applied to remove image artifacts at regions outside the larvae. All processing steps were performed in MATLAB (MathWorks Inc., Natick, MA, United States).

### Widefield epifluorescence recordings

Widefield epi-FL images of GCaMP5G signals were simultaneously captured with a high speed EMCCD camera (iXon Life 888, Andor Technology, Belfast, United Kingdom) having a 105 mm F-mount objective (Nikon, Chiyoda, Tokyo, Japan). FL emission was recorded using a longpass filter with a cut-on wavelength of 500 nm, effectively filtering the 488 nm excitation wavelength also used for OAT imaging (Figure 1C). The camera was manually adjusted so that the zebrafish larvae lie in focus and synchronized with the pulsed laser source. Much like for the volumetric OAT recordings, differential FL signal changes with respect to the baseline [ΔF/F_0_ = (F−F_0_)/F_0_] were calculated from the same regions in the hind and forebrain. The baseline value (F_0_) was also calculated by averaging the first ten time points at the beginning of the acquired FL sequence.

### Epileptic seizure propagation speed

Propagation speed of epileptic seizures was estimated by first identifying the regions where the seizure initiates (tail or hindbrain) and where it ends (forebrain). This was achieved by considering the time points corresponding to the peak values of the differential OA and FL signals. Propagation speeds (μm/s) were then calculated for several zebrafish larvae by considering the distance between the identified start and end points and the corresponding time interval.

## Results and discussion

The capability of the OAT system to distinguish internal structures was first assessed by comparing between the concurrently acquired OAT and FL images of an unrestricted 5 dpf control zebrafish larva (*eaat2a*^+/+^ or ^+/–^). The overall shape of the fish visualized with both modalities matches well, particularly when considering the top projection view of the volumetric OA image (Figure 1B, left). The eyes of the larva strongly contrast in the OAT images due to the high absorption by melanin. Other prominent structures such as the spinal cord are also visible. A better anatomical differentiation is achieved by the frequency band separation with the coarser structures such as the eyes emphasized in the green-colored 0.1–5 MHz band whilst finer structures of the nervous system, including the brain and the spinal cord, being accentuated within the high (5–25 MHz) frequency band (Figure 1D).

The feasibility of non-invasive visualization of the initiation and propagation of the epileptic seizure with the OAT system was further demonstrated with a 5 dpf *eaat2a*^–/–^ mutant zebrafish larva (Figure 2). The seizure was optically excited with a short-pulsed laser tuned to 488 nm, which further served to excite calcium-related OAT and FL responses at the peak absorption wavelength of GCaMP5G. A mechanical shutter was opened 10 s after starting the laser to ensure that the per-pulse laser energy remained stable for all the acquired frames. Signal changes over the first 50 s from the beginning of the acquisition were observed with both modalities (Figures 2A,B). The differential time-lapse OA and FL signal profiles from selected points in the hindbrain and forebrain are shown to approximately match (Figure 2C). The decrease in OA and FL signals in the hindbrain with respect to the initial values is ascribed to the fact that seizure activity had already started before acquisition and was declining in the hindbrain while propagating through the forebrain. On the other hand, a signal increase in OA and FL with respect to the initial values was observed in the forebrain at approximately 12 s after the beginning of the acquisition. This was followed by a decrease in OA and FL signals with respect to the initial value at the end of seizure propagation.

Neuronal activation maps for *eaat2a*^–/–^ mutant zebrafish larvae exposed to 488 nm light were further built from the acquired OAT image sequence. For this, the relative OA signal changes (ΔOA/OA_0_) were superimposed onto the normalized (between 0 and maximum value) OA images at five selected time points, namely, 0, 3.2, 13.8, 20, and 42.2 s after the beginning of light-induced stimulation (Figure 3A). Both images were plotted in different colormaps, and a transparency map was applied to the image representing the activity map (relative OA changes). The relative OA signal changes across the zebrafish body exceeded 100%. These generally represent the relative changes in calcium signals corresponding to neuronal activity, although some baseline OA signal level exists that corresponds to background tissue absorption. Particularly, the measured relative changes may be smaller than the actual change in the GCaMP5G absorption, especially in the regions around the eyes where the background absorption is very high. As the epileptic seizure propagates, calcium signals are shown to increase in the spinal cord eventually reaching the tail fin. The spatio-temporal neuronal activation patterns from the hindbrain (where the seizure originated) toward the forebrain are also observed in the corresponding FL images (Figure 3B). Five regions of interest (ROIs) indicated with different colored circles (blue, orange, yellow, purple, and green) in Figure 3B were selected to better analyze the spatio-temporal signal profiles associated to the induced seizure. The peak signal for the selected points was shown to vary between 3 and 8 s after the beginning of light-induced stimulation (black dashed lines in Figure 3C). The measured propagation speed of the seizure was 44.1 μm/s. Propagation speed measurements in FL and OA images were repeated for other *eaat2a*^–/–^ mutant zebrafish larvae (5 dpf, *n* = 7), represented with boxplots indicating the median, 25th and 75th percentiles in Figure 3D. The average seizure propagation speeds calculated from FL and OA images were 39.6, ± σ [58.6001, 20.5713] and 43.5, ± σ [64.3477, 22.5951] μm/s, respectively, indicating that the spatio-temporal resolution achieved with the OAT system is sufficient for accurately measuring the seizure propagation speed.

Light-induced epileptic seizures further caused aberrant swimming activity in some larvae captured with OA (Figure 4A) and FL (Figure 4B) images. It has been previously demonstrated that *eaat2a*^–/–^ larvae are less active between epileptic episodes compared to their *eaat2a*^+/–^ and *eaat2a*^+/+^ siblings (Hotz et al., 2022). However, *eaat2a*^–/–^ larvae tend to perform repetitive swimming in a circular direction (circling) during the epileptic seizures (Kalueff et al., 2013). The calcium signal propagation shown in Figure 2 was observed following aberrant swimming activity and circling (Supplementary Video 1), Note that non-invasive FL monitoring of neuronal activity during free behavior is significantly challenged by the limited depth of focus of the optical system. Whilst relatively high-resolution FL images could be collected for the fish lying in the optical focal plane, the resolution strongly deteriorates once it moves along the vertical direction (Figure 4A). Therefore, calcium signal changes cannot be accurately collected within a large 3D region with the image intensity varying significantly in different axial planes (depths). Recently, GCaMP imaging in freely-swimming zebrafish larvae achieved with the state-of-the-art optical methods by constraining the vertical motion only to few hundreds of μm (Kim et al., 2017; Zhang et al., 2021). On the other hand, volumetric OAT images exhibit isotropic 3D spatial resolution and nearly constant sensitivity at various depths (Figure 4B).

Finally, we analyzed different types of behavioral responses to the light-induced stimulation paradigm applied to the zebrafish larvae. In general, four different responses were observed following the 488 nm light stimulation, including zebrafish larval motion (circling, swimming) along with neuronal activation (m + a +), no motion or activation (m − a −), only motion without activation (m + a −) and only activation without motion (m − a +). Overall, 67 stimulations were induced on 12 *eaat2a*^–/–^ mutant zebrafish larvae. From this, 16 (23.8%) belonged to m + a +, while 41 (61.2%), 3 (4.5%), and 7 (10.5%) of them belonged to m − a−, m + a − and m − a + response groups, respectively (Figure 4C). We observed 100% increase of neuronal activity along with circling/swimming motion in *eaat2a*^–/–^ mutant zebrafish larvae.

## Conclusion

In this work, we demonstrated that neuronal activity and associated behavioral changes in zebrafish larvae undergoing epileptic seizures can be captured with volumetric OAT imaging. The high temporal resolution enabled the visualization of fast calcium dynamics in real-time, independently validated with simultaneously recorded widefield FL images. The capability of tracking neuronal activation in unrestrained animals is expected to provide a better understanding of the link between neuronal activity and behavioral changes during epileptic seizures, thus providing new insights on the basic mechanisms of epilepsy and paving the way toward development of more effective anti-epileptic drugs.

## Data availability statement

The original contributions presented in this study are included in the article/Supplementary material, further inquiries can be directed to the corresponding author.

## Ethics statement

This animal study was reviewed and approved by Cantonal Veterinary Office of Zurich (BNr-ZH150).

## Author contributions

ÇÖ: design of experiments, data collection, analysis, and interpretation of data, and writing the manuscript. AH: design of experiments, data collection, and writing the manuscript. NR and ZC: data collection. XD-B: data collection and revising the manuscript. SN and DR: design of experiments and revising the manuscript. All authors contributed to the article and approved the submitted version.
